# Prevalence of minimal hearing loss in South Korea

**DOI:** 10.1371/journal.pone.0171635

**Published:** 2017-02-14

**Authors:** Ji Eun Choi, Jungmin Ahn, Hyun Woo Park, Sun-Young Baek, Seonwoo Kim, Il Joon Moon

**Affiliations:** 1 Department of Otorhinolaryngology - Head and Neck Surgery, Samsung Medical Center, Sungkyunkwan University School of Medicine, Seoul, Republic of Korea; 2 Biostatistics and Clinical Epidemiology Center, Research Institute for Future Medicine, Samsung Medical Center, Sunkyunkwan University School of Medicine, Seoul, Republic of Korea; Fu Jen Catholic University, TAIWAN

## Abstract

This study evaluated the prevalence of minimal hearing loss (MHL) in South Korea based on the 2010 to 2012 Korea National Health and Nutrition Examination Survey. A total of 16,630 representative individuals (older than 12 years) who completed ear examinations and structured questionnaires were analyzed. Only participants who had normal tympanic membranes were included. MHL was categorized into the following three groups: 1) unilateral sensorineural hearing loss (USHL, pure-tone average (PTA) ≥ 15 dB in the affected ear), 2) bilateral sensorineural hearing loss (BSHL, 15 dB ≤ PTA < 40 dB in both ears), and 3) high-frequency sensorineural hearing loss (HFSHL, two or more high-frequency thresholds > 25 dB in either ear). To evaluate clinical symptoms, subjective hearing status, tinnitus, and quality of life of each MHL group were compared to those of normal-hearing listeners. The use of hearing aids (HAs) was also investigated in the MHL population. The prevalence of normal hearing and MHL were 58.4% and 37.4%, respectively. In univariate analyses, the prevalence of MHL increased with age. It was significantly increased in males. Regarding clinical symptoms, 13.0% and 92.1% of participants with MHL reported difficulties with hearing and annoying tinnitus, respectively. In multivariate analyses, these proportions were significantly higher in the MHL groups than in normal-hearing listeners. Participants with MHL also showed significantly lower Euro Qol-5D index scores than did normal-hearing listeners. Regarding hearing rehabilitation, among minimally hearing impaired participants with subjective hearing loss, only 0.47% of individuals used HAs. Our results reveal that MHL is common in South Korea. It is associated with significant subjective hearing loss, tinnitus, and poor quality of life. Therefore, clinicians need to pay attention to this special group and provide proper counselling and rehabilitative management.

## Introduction

The prevalence of hearing impairment is increasing owing to an aging society and growing use of personal listening devices [1, 2]. Hearing impaired individuals experience decreased hearing ability, reduced dynamic range, lower frequency resolution, reduced temporal resolution, and increased listening fatigue. Hearing impairment can limit their communication and social activity [3], leading to a lower quality of life and decreased cognitive function [4–6]. The majority of surveys to date have covered only bilateral hearing loss greater than 40 dB HL because of insufficient evidence regarding the effectiveness of interventions. However, individuals with minimal or mild bilateral hearing loss and high frequency hearing loss may experience difficulty understanding speech under adverse listening conditions. Unilateral hearing loss can also predispose individuals to reduced hearing ability and increased listening fatigue.

In an earlier study, Bess et al. (1998) categorized minimal hearing loss (MHL) into three distinct groups (mild bilateral hearing loss, unilateral hearing loss, and high-frequency hearing loss) and demonstrated an association between MHL and educational performance and functional status in school-aged children [7, 8]. Although the definition of MHL differs depending on the source, previous studies have demonstrated that children with MHL are at risk for greater academic, speech-language, and social-emotional difficulties than are their normal hearing peers [7–9]. Adults with MHL can also experience less satisfaction and reduced emotional well-being than do normal hearing individuals [9, 10]. Despite this concern, only a few studies have used audiometric testing to gauge the demographic characteristics and associated symptoms of MHL at the national level [11–13].

Thus, the objective of this study was to determine the prevalence of MHL in South Korea based on national survey data obtained from the 2010 to 2012 Korea National Health and Nutrition Examination Survey (KNHANES) and assess the quality of life of people with MHL. The definition of MHL used in this study was based on the previous study by Bess et al. (1998).

## Methods

### Study population and data collection

This study used the data from the fifth KNHANES. The KNHANES is a nationwide survey conducted annually by the Korea Centers for Disease Control and Prevention to investigate the health and nutritional status of a representative Korean population. Every year, about 10,000 individuals in 3,840 households are selected from a panel to represent the population through a multistage clustered and stratified random sampling method based on the National Census Data. A total of 576 survey areas were drawn from the population and housing census by considering the proportion of each subgroup. The participation rate of selected households was about 80%. From 2010 to 2012, a total of 23,621 individuals (8,313 in 2010, 7,887 in 2011, and 7,421 in 2012) agreed to participate in the health surveys. They underwent ear, nose, and throat (ENT) examinations. To exclude mixed or conductive hearing loss, individuals with tympanic membrane perforation and cholesteatomatous conditions including retraction pocket, otitis media with effusion, and insertion of a ventilation tube were excluded. Among 19,864 participants who had normal tympanic membranes, 16,630 participants completed both the audiometric measurement and the ENT questionnaire.

All participants provided written informed consent before completing the survey. KNHANES followed the tenets of the Declaration of Helsinki for biomedical research. It was approved by the Institutional Review Board of the Korean Centers for Disease Control and Prevention (IRB No. 2010-02CON-21-C, 2011-02CON-06-C, and 2012-01EXP-01-2C). Written informed consent was also obtained from the next of kin, caretakers, or guardians of minors/children enrolled in this survey. Approval for this research study was obtained from the Institutional Review Board of Samsung Medical Center (IRB No. 2016-02-076).

### Audiometric measurement and otologic examination

Pure tone threshold was measured in a sound-proof booth using an automatic audiometer (GSI SA-203, Entomed Diagnosics AB, Lena Nodin, Sweden). Otolaryngologists who had been trained to operate the audiometer provided instructions to participants and obtained recordings. Audiometry was performed for participants over 12 years of age. Only air conduction thresholds were measured. Supra-auricular headphones were used in the soundproof booth. The otolaryngologist provided basic instructions to participants regarding the automated hearing test. Automated testing was programmed using a modified Hughson-Westlake procedure with a single pure tone for 1–2 seconds. The lowest pure tone level at which the subject’s response rate was 50% was set as the threshold. Participants responded by pushing a button when they heard a tone. Results were automatically recorded. The following frequencies were tested: 0.5, 1, 2, 3, 4, and 6 kHz. An ear examination was conducted with a 4 mm 0°-angled rigid endoscope attached to a Charge-Coupled Device (CCD) camera by trained otolaryngologists. Endoscopic examination was performed to identify tympanic membrane perforation, cholesteatoma (including retraction pocket), and otitis media with effusion (including the presence of a ventilation tube).

### Definition of minimal hearing loss

Minimal sensorineural hearing loss was categorized into three distinct groups according to *Bess et al*. (1998): unilateral sensorineural hearing loss (USHL), bilateral sensorineural hearing loss (BSHL), and high-frequency sensorineural hearing loss (HFSHL) [8]. USHL was defined as average air-conduction thresholds (0.5, 1, and 2 kHz) ≥ 15 dB HL in the affected ear and < 15 dB HL in the unaffected ear. USHL was subdivided into slight hearing loss and mild-to-profound loss. Slight USHL was defined as average air-conduction thresholds < 25 dB HL. Mild-to-profound USHL was defined as ≥ 25 dB HL. BSHL was defined as average air-conduction thresholds (0.5, 1, and 2 kHz) between 15 and 40 dB HL bilaterally. BSHL was subdivided into minimal BSHL and mild BSHL. Minimal BSHL was defined as average air-conduction thresholds between 15 and 25 dB HL bilaterally. Mild BSHL was defined as average air-conduction thresholds > 25 dB HL bilaterally. HFSHL was defined as air-conduction thresholds greater than 25 dB HL at two or more frequencies above 2 kHz (i.e., 3, 4, 6 kHz) in one or both ears. Those with HFSHL had normal hearing (< 15 dB HL) at 0.5, 1, and 2 kHz in both ears. HFSHL was subdivided into unilateral HFSHL and bilateral HFSHL. Normal hearing was defined as average air-conduction thresholds (0.5, 1, and 2 kHz) < 15 dB HL and less than 25 dB HL in both ears at two or more frequencies above 2 kHz. Moderate hearing loss was defined as average air-conduction thresholds (0.5, 1, and 2 kHz) > 40 dB HL in either one ear or both ears.

### Outcome variables

To determine clinical symptoms, participants completed a questionnaire asking about their hearing and whether they had any symptoms of tinnitus. Subjective hearing status was measured by asking the following survey question: “Which sentence best describes your hearing status (while using no HAs)?”. There were four answers for the question: (1) “Don’t feel difficulty at all,” (2) “A little bit difficult,” (3) “Very difficult,” and (4) “Can’t hear at all.” Subjective hearing loss was indicated when the response was (2), (3), or (4). Participants were also asked about their experience with tinnitus. In response to the question “Within the past year, did you ever hear a sound (buzzing, hissing, ringing, humming, roaring, machinery noise) originating in your ear?”, examiners were instructed to record “yes” if a participant reported that they heard an odd or unusual noise at any time in the past year. Participants who responded positively to this question were then queried concerning the resulting annoyance in their lives using the following questions: “How severe is this noise in daily life?” (not annoying, annoying, severely annoying, or causing sleep problems). Participants were assigned to the group with annoying tinnitus if the severity of tinnitus was annoying or severely annoying. Regarding quality of life, the Euro Qol-5D (EQ-5D) was used to evaluate all participants aged 18 years or older. The EQ-5D is a standard tool used to measure patients’ health status in the following five dimensions: mobility, self-care, usual activities, pain/discomfort, and anxiety/depression [14, 15]. Each dimension has three grades of severity: no problem (score of 1), moderate problem (score of 2), or serious problem (score of 3). The EQ-5D index is calculated from the EQ-5D score by applying a formula that assigns weights to each grade in each dimension. This formula differs among nations because it is based on the value of the EQ-5D of the population sample [16]. The KNHANES algorithm was used to calculate the EQ-5D index in this study. The EQ-5D index ranged from 1 (best health) to 0 (equivalent to death) or -0.171 (worse than death). To evaluate hearing rehabilitation for MHL, participants were asked about their use of HAs. Responses to the question of “Do you currently use any HAs?” included “yes,” “yes, but rarely,” “no,” and “not applicable.” When participants reported having “no difficulty” with their hearing, the use of an HA was considered to be “not applicable.”

### Statistical analysis

All statistical analyses were performed by taking into account the weights from the complex sampling design according to the guidelines for analysis of KNHANES data obtained by the Korea Centers for Disease Control and Prevention. The survey design created a sample weight assigned to each sample individual through the following three steps so that the total sample would represent the population (on average) for the 3-year period (2010–2012): calculating the base weight of the inverse of the final probability of an individual being selected, adjusting for non-response, and post-stratification adjustment to match previous census population control totals. The weights in the 2010, 2011, and 2012 surveys were combined and the average weight (weight for each year/3) was calculated. Statistical analyses were performed using SAS version 9.4 (SAS Institute, Cary, NC, USA). The prevalence of MHL was then estimated. The chi-squared test was used to compare the prevalence of MHL among age groups and according to sex. Logistic regression or linear regression was used to compare the prevalence of MHL to normal hearing according to the responses to the questionnaires. *P*-values were two-sided. Bonferroni’s correction was applied to *P*-values and the corresponding confidence intervals owing to multiple testing. Statistical significance was considered when an adjusted *P*-value was less than 0.05.

## Results

### Prevalence of minimal hearing loss

Of 16,630 participants, 58.4% had normal hearing, while 37.4% had MHL (Table 1). BSHL accounted for the highest proportion (42.8%) of MHL, followed by USHL (37.5%) and HFSL (19.7%). Among participants with USHL, most participants (80.1% of those with USHL) had slight hearing loss. Among those with HFSHL, both ears were affected in 57.2% of cases, while 42.8% of individuals were affected unilaterally.

**Table 1:** Prevalence of minimal hearing loss and its subgroups.

Classification	Frequency	Weighted Frequency	Weighted Percent (%)
**Normal hearing**	8,511	20,908,897	58.41
**Minimal hearing loss**	7,058	13,373,850	37.36
**USHL**	2,443	5,009,016	13.99
Slight USHL	1,949	4,010,785	11.2
Mild to profound USHL	494	998,231	2.79
**BSHL**	3,426	5,724,301	15.99
Minimal BSHL	1,868	3,323,898	9.29
Mild BSHL	1,558	2,400,402	6.71
**HFSHL**	1,189	2,640,533	7.38
Unilateral HFSHL	528	1,130,163	3.16
Bilateral HFSHL	661	1,510,370	4.22
**≥ Moderate hearing loss**	1,061	1,513,466	4.23
**Total**	16,630	35,796,213	100

USHL, unilateral sensorineural hearing loss; BSHL, bilateral sensorineural hearing loss; HFSHL, high-frequency sensorineural hearing loss

**Fig 1:**
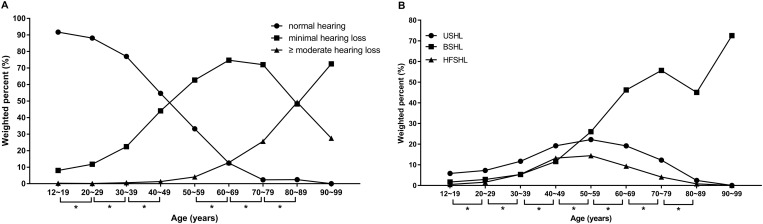
Prevalence of minimal hearing loss and its subgroups according to age. (A) The prevalence of minimal hearing loss was significantly different between age groups in post-hoc analysis, except among subjects in their forties and fifties. (B) The prevalence of all subgroups of minimal hearing loss were significantly different between age groups in post-hoc analysis. Chi-squared analysis revealed that the prevalence of minimal hearing loss was significantly different between age groups (*P* < 0.001). An asterisk (*) indicates a significant difference after adjustment using Bonferroni's method. USHL: unilateral sensorineural hearing loss; BSHL: bilateral sensorineural hearing loss; HFSHL: high-frequency sensorineural hearing loss.

The prevalence of MHL according to nine different age groups is demonstrated in Fig 1. The prevalence of MHL significantly (*P* < 0.0001) differed among age groups based on chi-squared testing and post-hoc analysis, except amongst those in their forties (40 to 49 years of age) or fifties (50 to 59 years of age). The prevalence of MHL increased with age until the 6th decade of life (60 to 69 years of age) and decreased afterwards (Fig 1A). Regarding subcategories of MHL, the prevalence of USHL and HFSHL increased until the 5th decade of life. They then decreased with age. However, the prevalence of BSHL increased with age until the 7th decade of life (70 to 79 years of age) (Fig 1B).

The prevalence of MHL and its subgroups according to sex are shown in Table 2. MHL was predominant in males (41.4%) compared to females (33.3%). HFSHL (unilaterally or bilaterally) was especially prevalent in males (12.2%) (Table 2). When the prevalence of HFSHL was compared among different age groups, HFSHL was also prevalent in males except in those over 70 years old (Table 3).

**Table 2:** Prevalence of minimal hearing loss and its subgroups according to sex.

Sex	Male			Female			*P*-value
Classification	Frequency	Weighted Frequency	Weighted Percent (%)	Frequency	Weighted Frequency	Weighted Percent (%)	
**Normal hearing**	3,310	9798698	54.64	5,201	11110198	62.2	< 0.0001*
**Minimal hearing loss**	3,441	7,427,722	41.42	3,617	5,946,128	33.29	< 0.0001*
**USHL**	1,059	2,495,340	13.91	1,384	2,513,676	14.07	0.8841
Slight USHL	861	2,045,206	11.4	1,088	1,965,579	11	0.4607
Mild to profound USHL	198	450,134	2.51	296	548,097	3.07	0.1189
**BSHL**	1,472	2,740,396	15.28	1,954	2,983,904	16.7	0.0443*
Minimal BSHL	792	1,615,053	9.01	1,076	1,708,845	9.57	0.3423
Mild BSHL	680	1,125,343	6.28	878	1,275,059	7.14	0.0584
**HFSHL**	910	2,191,985	12.22	279	448,548	2.51	< 0.0001*
Unilateral HFSHL	436	984,304	5.49	92	145,859	0.82	< 0.0001*
Bilateral HFSHL	474	1,207,681	6.73	187	302,689	1.69	< 0.0001*
**≥ Moderate hearing loss**	487	706,891	3.94	574	806,575	4.52	
**Total**	7,238	17,933,311	100	9,392	17,862,902	100	

The chi-squared test was used to compare the prevalence of minimal hearing loss and its subgroups according to sex. An asterisk (*) indicates a significant difference (P < 0.05).

USHL: unilateral sensorineural hearing loss; BSHL: bilateral sensorineural hearing loss; HFSHL: high-frequency sensorineural hearing loss

**Table 3:** Weighted frequency of minimal hearing loss and its subgroups according to sex and age.

Age groups	MHL		adjusted *P*-value	USNHL		adjusted *P*-value	BSHL		adjusted *P*-value	HFSHL		adjusted *P*-value
	Male	Female		Male	Female		Male	Female		Male	Female	
**12 ~ 19**	177,038	182,998	1.000	112,425	149,661	1.000	43,407	32,379	1.000	21,206	958	<.0001*
**20 ~ 29**	399,892	298,119	0.3672	200,211	230,954	1.000	112,951	58,831	0.835	86,730	8,334	<.0001*
**30 ~ 39**	978,898	567,682	<.0001*	414,671	391,691	1.000	220,475	146,791	0.982	343,752	29,200	<.0001*
**40 ~ 49**	1,964,030	1,176,991	<.0001*	740,602	629,625	1.000	421,471	405,385	1.000	801,956	141,980	<.0001*
**50 ~ 59**	2,036,032	1,538,692	<.0001*	608,902	658,986	1.000	760,143	723,634	1.000	666,987	156,072	<.0001*
**60 ~ 69**	1,195,088	1,179,316	<.0001*	302,672	306,327	1.000	675,283	793,970	0.449	217,133	79,019	<.0001*
**70 ~ 79**	603,489	882,917	<.0001*	110,878	142,028	1.000	441,461	707,906	<.0001*	51,149	32,984	1.000
**80~**	72,993	114,174	0.4192	4,978	4,403	1.000	64,942	109,771	0.744	-	-	-

The chi-squared test was used to compare the prevalence of minimal hearing loss and its subgroups according to sex and age. An asterisk (*) indicates a significant difference (adjusted P < 0.05).

MHL: Minimal sensorineural hearing loss; USHL: unilateral sensorineural hearing loss; BSHL: bilateral sensorineural hearing loss; HFSHL: high-frequency sensorineural hearing loss

### Normal hearing versus minimal hearing loss

After excluding participants who had worse than moderate hearing loss (PTA > 40 dB HL either in one ear or both ears), a total of 15,569 participants were analyzed to compare the prevalence of MHL to that of normal hearing using linear and logistic regression analyses. In univariate analyses, the prevalence of MHL increased with age (*P* < 0.0001, OR: 1.098, 95% CI: 1.093–1.102). It was higher in males (*P* < 0.0001, OR: 1.416, 95% CI: 1.307–1.534) (Table 4). The prevalence of the subgroups of MHL also increased with age, especially for BSHL (*P* < 0.0001, OR: 1.135, 95% CI: 1.124–1.146). However, sex was associated with the prevalence of MHL only for HFSHL. HFSHL was significantly predominant in males (*P* < 0.0001, OR: 5.541, 95% CI: 4.478–6.857). The prevalence of MHL in the HFSHL subgroup was also higher in males, regardless of whether the ear was affected unilaterally (*P* < 0.0001, OR: 7.651, 95% CI: 5.138–11.392) or bilaterally (*P* < 0.0001, OR: 4.524, 95% CI: 3.396–6.027).

**Table 4:** Univariate analyses of age and sex in the minimal hearing loss group compared to the normal hearing group.

**Age**	**Frequency**	**Weighted Frequency**	**Average (years)**	***P*-value**	**OR**	**95% CI**
**Classifications**						
**Total**	15,569	34,282,747	35.16	-	-	-
**Normal hearing (ref)**	8,511	20,908,897	32.52	ref	ref	ref
**Minimal hearing loss**	7,058	13,373,850	51.62	< 0.0001*	1.098	1.093–1.102
**USHL**	2,443	5,009,016	46.19	< 0.0001*	1.074	1.067–1.082
Slight USHL	1,949	4,010,785	46.67	< 0.0001*	1.078	1.070–1.087
Mild to profound USHL	494	998,231	44.24	< 0.0001*	1.064	1.046–1.082
**BSHL**	3,426	5,724,301	57.91	< 0.0001*	1.135	1.124–1.146
Minimal BSHL	1,868	3,323,898	54.36	< 0.0001*	1.120	1.109–1.131
Mild BSHL	1,558	2,400,402	62.81	< 0.0001*	1.180	1.158–1.203
**HFSHL**	1,189	2,640,533	48.3	< 0.0001*	1.096	1.089–1.104
Unilateral HFSHL	528	1,130,163	51.19	< 0.0001*	1.114	1.102–1.126
Bilateral HFSHL	661	1,510,370	46.14	< 0.0001*	1.08	1.071–1.090
**Sex**	**Frequency**	**Weighted Frequency**	**Weighted Percent (%)**	***P*-value**	**OR**	**95% CI**
**Classifications**						
**Total**						
male	6,751	17,226,420	50.25	-	-	-
female	8,818	17,056,327	49.75			
**Normal hearing (ref)**						
male	3,310	9,798,698	46.86	ref	ref	ref
female	5,201	11,110,198	53.14			
**Minimal hearing loss**						
male (ref)	3,441	7,427,722	55.54	< 0.0001*	1.416	1.307–1.534
female	3,617	5,946,128	44.46			
**USHL**						
male (ref)	1,059	2,495,340	49.82	0.1479	1.126	0.975–1.299
female	1,384	2,513,676	50.18			
**Slight USHL**						
male (ref)	861	2,045,206	50.99	0.0516	1.18	1.000–1.392
female	1,088	1,965,579	49.01			
**Mild to profound USHL**						
male (ref)	198	450,134	45.09	1.000	0.931	0.659–1.316
female	296	548,097	54.91			
**BSHL**						
male (ref)	1,472	2,740,396	47.87	1.000	1.041	0.923–1.175
female	1,954	2,983,904	52.13			
**Minimal BSHL**						
male (ref)	792	1,615,053	9.01	1.000	1.072	0.900–1.276
female	1,076	1,708,845	9.57			
**Mild BSHL**						
male (ref)	680	1,125,343	6.28	1.000	1.001	0.831–1.206
female	878	1,275,059	7.14			
**HFSHL**						
male (ref)	910	2,191,985	83.01	< 0.0001*	5.541	4.478–6.857
female	279	448,548	16.99			
**Unilateral HFSHL**						
male (ref)	436	984,304	87.09	< 0.0001*	7.651	5.138–11.392
female	92	145,859	12.91			
**Bilateral HFSHL**						
male (ref)	474	1,207,681	79.96	< 0.0001*	4.524	3.396–6.027
female	187	302,689	20.04			

An asterisk (*) indicates a significant difference (P < 0.05).

USHL, unilateral sensorineural hearing loss; BSHL, bilateral sensorineural hearing loss; HFSHL, high-frequency sensorineural hearing loss; CI, confidence interval; OR, odds ratio

Comparisons of participants with MHL and normal hearing regarding subjective complaints of hearing loss and tinnitus are summarized in Table 5. Compared to normal hearing participants, the proportion of participants complaining about their hearing difficulties was significantly higher in participants with MHL (*P* < 0.0001, OR: 2.729, 95% CI 2.217–3.360) after adjusting for age and sex. While 13.0% of participants with MHL reported difficulties with their hearing, only 3.1% of participants with normal hearing reported subjective hearing loss. In multivariate analyses, participants with MHL also complained of tinnitus significantly more than did normal hearing participants (*P* < 0.0001, OR: 1.520, 95% CI: 1.333–1.734). Annoying tinnitus was observed significantly more often in participants with MHL compared to those with normal hearing (*P* < 0.0001, OR: 1.868, 95% CI: 1.511–2.310). In the subgroups, participants with mild-to-profound USHL complained of their hearing difficulties the most (*P* < 0.0001, OR: 6.556, 95% CI: 3.904–11.010), followed by participants with mild BSHL (*P* < 0.0001, OR: 6.352, 95% CI: 4.229–9.540). Regarding tinnitus, participants with mild BSHL mostly reported tinnitus (*P* < 0.0001, OR: 3.145, 95% CI: 2.362–4.189), while participants with mild-to-profound USHL mostly reported annoying tinnitus (*P* < 0.0001, OR: 3.906, 95% CI: 2.277–6.701).

**Table 5:** Univariate and multivariate analyses of clinical symptoms in the minimal hearing loss group compared to the normal hearing group.

**Classifications**	**Frequency**	**Weighted Frequency**	**Weighted Percent (%)**	**Univariable analysis**			**Multivariable analysis**		
				***P*-value**	**OR**	**95% CI**	***P*-value**	**OR**	**95% CI**
**Subjective hearing loss**									
**Total**									
not discomfort	14,245	31,889,494	93	-	-	-	-	-	-
discomfort	1,324	2,393,253	7						
**Normal hearing (ref)**									
not discomfort	8,243	20,255,980	96.9	ref	ref	ref	ref	ref	ref
discomfort	268	652,917	3.1						
**Minimal hearing loss**									
not discomfort	6,002	11,633,514	87	< 0.0001*	4.641	3.901–5.522	< 0.0001*	2.729	2.217–3.360
discomfort (event)	1,056	1,740,336	13						
**USHL**									
not discomfort	2,178	4,515,893	90.2	< 0.0001*	3.388	1.599–2.119	< 0.0001*	2.484	1.815–3.400
discomfort (event)	265	493,123	9.8						
**Slight USHL**									
not discomfort	1,796	3,729,636	93	< 0.0001*	2.339	1.258–1.859	0.0168*	1.61	1.059–2.447
discomfort (event)	153	281,149	7						
**Mild to profound USHL**									
not discomfort	382	786,257	78.8	< 0.0001*	8.365	2.266–3.692	< 0.0001*	6.556	3.904–11.010
discomfort (event)	112	211,974	21.2						
**BSHL**									
not discomfort	2,738	4,672,983	81.6	< 0.0001*	6.98	2.360–2.957	< 0.0001*	3.781	2.768–5.166
discomfort (event)	688	1,051,318	18.4						
**Minimal BSHL**									
not discomfort	1,625	2,923,129	87.9	< 0.0001*	4.254	1.782–2.388	< 0.0001*	2.687	1.782–4.051
discomfort (event)	243	400,770	12.1						
**Mild BSHL**									
not discomfort	1,113	1,749,854	72.9	< 0.0001*	11.534	2.957–3.901	< 0.0001*	6.352	4.229–9.540
discomfort (event)	445	650,548	27.1						
**HFSHL**									
not discomfort	1,086	2,444,638	92.6	< 0.0001*	2.486	1.316–1.889	0.0141*	1.732	1.090–2.752
discomfort (event)	103	195,895	7.4						
**Unilateral HFSHL**									
not discomfort	466	1,004,601	88.9	< 0.0001*	3.878	1.529–2.537	< 0.0001*	2.499	1.351–4.622
discomfort (event)	62	125,562	11.1						
**Bilateral HFSHL**									
not discomfort	620	1,440,037	95.3	0.3012	1.515	0.931–1.628	1.000	1.089	0.563–2.108
discomfort (event)	41	70,333	4.7						
**Presence of tinnitus**									
**Total**									
yes	3,120	6,679,347	19.5	-	-	-	-	-	-
no	12,449	27,603,399	80.5						
**Normal hearing (ref)**									
yes	1,435	3,616,316	17.3	ref	ref	ref	ref	ref	ref
no	7,076	17,292,580	82.7						
**Minimal hearing loss**									
yes (event)	1,685	3,063,031	22.9	< 0.0001*	1.421	1.275–1.583	< 0.0001*	1.520	1.333–1.734
no	5,373	10,310,819	77.1						
**USHL**									
yes (event)	527	1,065,106	21.3	0.0018*	1.291	1.040–1.242	< 0.0001*	1.460	1.199–1.776
no	1,916	3,943,910	78.7						
**Slight USHL**									
yes (event)	393	798,167	19.9	0.2598	1.188	0.974–1.219	0.0042*	1.376	1.075–1.762
no	1,556	3,212,618	80.1						
**Mild to profound USHL**									
yes (event)	134	266,939	26.7	0.0002*	1.745	1.108–1.575	< 0.0001*	2.010	1.388–2.912
no	360	731,292	73.3						
**BSHL**									
yes (event)	906	1,462,309	25.5	< 0.0001*	1.641	1.184–1.386	< 0.0001*	1.939	1.580–2.378
no	2,520	4,261,991	74.5						
**Minimal BSHL**									
yes (event)	490	756,671	31.5	0.0216*	1.289	1.012–1.273	< 0.0001*	1.637	1.260–2.126
no	1,068	1,643,731	68.5						
**Mild BSHL**									
yes (event)	252	535,615	20.3	< 0.0001*	2.201	1.343–1.639	< 0.0001*	3.145	2.362–4.189
no	937	2,104,918	79.7						
**HFSHL**									
yes (event)	252	535,615	20.3	0.1653	1.217	0.976–1.246	< 0.0001*	1.871	1.402–2.496
no	937	2,104,918	79.7						
**Unilateral HFSHL**									
yes (event)	115	227,892	20.2	1	1.208	0.914–1.322	< 0.0001*	2.006	1.315–3.060
no	413	902,271	79.8						
**Bilateral HFSHL**									
yes (event)	137	307,723	20.4	0.7446	1.224	0.931–1.315	0.0004*	1.784	1.224–2.600
no	524	1,202,647	79.6						
**Presence of annoying tinnitus**									
**Total**									
yes	14,688	32,548,170	94.9	-	-	-	-	-	-
no	881	1,734,576	5.1						
**Normal hearing (ref)**									
yes	8,225	20,231,329	96.8	ref	ref	ref	ref	ref	ref
no	286	677,568	3.2						
**Minimal hearing loss**									
yes (event)	6,463	12,316,841	92.1	< 0.0001*	2.562	2.129–3.083	< 0.0001*	1.868	1.511–2.310
no	595	1,057,008	7.9						
**USHL**									
yes (event)	2,266	4,650,684	92.8	< 0.0001*	2.301	1.308–1.759	< 0.0001*	1.946	1.413–2.680
no	177	358,332	7.2						
**Slight USHL**									
yes (event)	1,833	3,781,937	94.3	0.0002*	1.807	1.117–1.618	0.0186*	1.545	1.050–2.274
no	116	228,848	5.7						
**Mild to profound USHL**									
yes (event)	433	868,748	87	< 0.0001*	4.45	1.626–2.736	< 0.0001*	3.906	2.277–6.701
no	61	129,484	13						
**BSHL**									
yes (event)	3,080	5,174,162	90.4	< 0.0001*	3.175	1.568–2.025	< 0.0001*	2.099	1.5523–2.894
no	346	550,139	9.6						
**Minimal BSHL**									
yes (event)	1,735	3,103,064	93.4	< 0.0001*	2.125	1.205–1.764	0.0210*	1.589	1.048–2.410
no	133	220,834	6.6						
**Mild BSHL**									
yes (event)	1,345	2,071,097	86.3	< 0.0001*	4.748	1.869–2.541	< 0.0001*	3.618	2.213–5.915
no	213	329,305	13.7						
**HFSHL**									
yes (event)	1,117	2,491,995	94.4	0.0018*	1.78	1.093–1.629	0.00451*	1.952	1.183–3.2219
no	72	148,538	5.6						
**Unilateral HFSHL**									
yes (event)	491	1,069,736	94.7	0.0774	1.687	0.985–1.712	0.162	1.734	0.901–3.336
no	37	60,427	5.3						
**Bilateral HFSHL**									
yes (event)	626	1,422,260	94.2	0.0324*	1.85	1.018–1.818	0.0288*	2.032	1.049–3.935
no	35	88,111	5.8						
**Classifications**	**Frequency**	**Weighted Frequency**	**Average**	**Univariable analysis**			**Multivariable analysis**		
				***P*-value**			***P*-value**		
**EQ-5D index**									
**Total**	13,730	29,912,914	0.956	-			-		
**Normal hearing (ref)**	6,906	17,066,487	0.972	ref			ref		
**Minimal hearing loss**	6,824	12,846,427	0.935	< 0.0001*			< 0.0001*		
**USHL**	2,311	4,716,951	0.949	< 0.0001*			0.0024*		
Slight USHL	1,850	3,785,067	0.949	< 0.0001*			0.0078*		
Mild to profound USHL	461	931,884	0.95	0.0001*			1.000		
**BSHL**	3,348	5,558,945	0.911	< 0.0001*			< 0.0001*		
Minimal BSHL	1,868	3,323,898	0.926	< 0.0001*			< 0.0001*		
Mild BSHL	1558	2,400,402	0.889	< 0.0001*			< 0.0001*		
**HFSHL**	1,165	2,570,532	0.962	0.0084*			0.936		
Unilateral HFSHL	520	1,099,964	0.961	0.168			1.000		
Bilateral HFSHL	645	1,470,568	0.962	0.1752			1.000		

Multivariate analysis adjusted for age and sex. An asterisk (*) indicates a significant difference (P < 0.05).

USHL, unilateral sensorineural hearing loss; BSHL, bilateral sensorineural hearing loss; HFSHL, high-frequency sensorineural hearing loss; CI, confidence interval; OR, odds ratio

A total of 13,730 participants completed the EQ-5D survey. Compared to the normal hearing group, the MHL group had a significantly (*P* < 0.0001) lower mean EQ-5D index score in the linear regression analysis after adjusting for age and sex. The average EQ-5D indices in the normal hearing and MHL groups were 0.972 and 0.935, respectively. In the subgroups, the mild BSHL group had the lowest average score on the EQ-5D (0.889), followed by the minimal BSHL group (0.926).

### Hearing rehabilitation in minimal hearing loss

The use of HAs among those with MHL is shown in Table 6. Among participants who suffered from subjective hearing loss, only 0.47% of minimally hearing impaired participants used HAs. Especially in the USHL and HFHL groups, hearing aids were hardly ever used. Among participants who reported subjective hearing loss, the percentage of hearing aid users did not differ significantly between participants with MHL and those with normal hearing (*P* = 0.2703, OR: 0.269, 95% CI: 0.026–2.785) in logistic regression analysis after adjusting for age and sex.

**Table 6:** The use of hearing aids by participants with minimal hearing loss.

Classification	Total	Subjective hearing		Use of hearing aids			Weighted percent (%)*
		No	Yes	Using	Rarely using	Not using	
	Frequency	Frequency	Frequency	Frequency	Frequency	Frequency	
**Total**	15,569	14,243	1,324	7	2	1,317	0.40
**Normal hearing (ref)**	8,511	8,243	268	1	0	267	0.21
**Minimal hearing loss**	7,058	6,002	1,056	6	2	1,048	0.47
**USHL**	2,443	2,178	265	0	0	265	0.00
Slight USHL	1,949	1,796	153	0	0	153	0.00
Mild to profound USHL	494	382	112	0	0	112	0.00
**BSHL**	3,426	2,738	688	5	2	681	0.66
Minimal BSHL	1,868	1,625	243	1	0	242	0.16
Mild BSHL	1,558	1,113	445	4	2	441	0.97
**HFSHL**	1,189	1,086	103	1	0	102	0.58
Unilateral HFSHL	528	466	62	1	0	61	0.90
Bilateral HFSHL	661	620	41	0	0	41	0.00

A questionnaire on the use of hearing aids was given to participants with subjective hearing loss. Weighted percent (%)* = number of current hearing aid users/number of participants with subjective hearing loss. USHL, unilateral sensorineural hearing loss; BSHL, bilateral sensorineural hearing loss; HFSHL, high-frequency sensorineural hearing loss; CI, confidence interval; OR, odds ratio

## Discussion

Using data from the KNHANES 2010–2012, we found that the weighted prevalence of MHL in the South Korean population aged 12 years or older was 37.4%. When MHL was divided into subgroups (USHL, BSHL, and HFSHL), the prevalence of USHL, BSHL, and HFSHL were 14%, 16%, and 7.4%, respectively. These prevalence rates are similar to those found in a previous survey, although there might be some differences in the definitions of hearing loss. The WHO prevalence statistics from 2012 were based on a definition of mild hearing loss as an average threshold at 0.5, 1, 2, and 4 kHz of between 26 and 40 dB HL [11]. The BSHL for all adults aged 15 years or older was calculated to be 9% to 17%, depending on geographic region. Based on the data from the US NHANES (2001–2008) study, USHL was defined as an average threshold at 0.5, 1, 2, and 4 kHz of greater than 25 dB HL in one ear [17]. The prevalence of USHL was reported to be 7.6% for participants aged 12 years or older [17].

In the present study, BSHL was the most prevalent category (48.5%), followed by USHL (34.6%) and HFSL (16.8%). Compared to a population-based study on Canadian children aged 0 to 18 years, the prevalence of each subgroup was slightly different. Although BSHL was the most prevalent category in both studies, USHL was more prevalent in adults aged 12 years or older than in children aged 18 years or younger (BSHL: 70%, bilateral HFSHL: 11.6%, and USHL: 18.4% in the Canadian study) [18]. This might be due to acquired unilateral hearing loss, such as sudden sensorineural hearing loss. The prevalence of MHL increased with age until participants were in their sixties. However, it decreased in those in their sixties to eighties (Fig 1A). The prevalence of USHL and HFSHL decreased with age after participants reached their fifties (Fig 1B). It is well known that the prevalence of hearing loss can rise sharply in adults over age 50 [12]. For that reason, subjects with HFSHL and USHL could be categorized into the moderate hearing loss group over time, especially after their fifties. Regarding sex, HFSHL was significantly more prevalent in males (Table 2), which is consistent with previous results [1, 2, 13].

The present study found that the proportion of participants complaining about their hearing with MHL was significantly higher than the proportion of normal hearing participants complaining about their hearing in univariate and multivariate analyses (Table 5). Participants with mild-to-profound USHL and mild BSHL complained of their hearing with a high odds ratio (OR: 6.556 for mild-to-profound USHL and 6.352 for mild BSHL, Table 5) compared to the normal hearing group after adjustment for age and sex. The minimally impaired hearing group also complained of annoying tinnitus significantly more than did the normal hearing group, especially participants with mild BSHL and mild-to-profound USHL (OR: 3.906 for mild-to-profound USHL and 3.618 for mild BSHL, Table 5). These observations emphasize the need to provide appropriate counseling to patients with MHL and to encourage them to consider communication strategies, assistive listening devices, or HAs. The use of assistive listening devices or HAs in patients with MHL may improve their communication by reducing the effort required for them to listen, particularly in noisy environments. Besides, HAs have been known to be effective for tinnitus-associated MHL by masking or distracting from tinnitus with amplified environmental sound [19, 20].

Despite suffering from subjective hearing loss, only 0.47% of participants with MHL reported that they currently use HAs (Table 6). A previous study reported that a total of 12.6% of subjects who had bilateral moderate-to-profound hearing loss (> 40dB hearing threshold measured at 0.5, 1, 2, and 3 kHz) and subjective hearing loss regularly used HAs [21]. Compared to those with bilateral moderate-to-profound hearing loss, participants with MHL rarely used HAs. Auditory rehabilitation has been directed toward remediating the disabilities or handicaps experienced by individuals who have hearing impairments at a hearing level greater than 30 to 40 dB [22]. There are some limitations to treating MHL, including cost, lack of insurance coverage, social stigma, and lack of engagement by health care providers. A number of consumer studies have suggested that, among an array of limitations, one factor responsible for the lower adoption of HAs by those with mild hearing loss might be clinicians themselves. The MarkeTrak survey in 2012 suggested that 29% of individuals who report mild hearing loss have discussed their hearing problems with an audiologist, 43% are advised to wait and retest, and 26% are told that HAs would not be beneficial [23]. One reason for advising against HAs may be that HAs are deemed less beneficial for those with mild hearing loss. Among patients with MHL for whom cost is a primary concern, personal sound amplification products (PSAP) could be a solution. According to a recently published article, a PSAP is a one-size fits all electronic device that can amplify soft sounds [24]. These devices were originally designed for normal hearing users to heighten their hearing ability for recreational activities. They can often be purchased at a low cost. Even though these products are not medically approved or recommended as a treatment option for permanent hearing loss, a PSAP can be a helpful, affordable, and accessible initial option for those with bilateral MHL. Regarding mild-to-profound USHL, bone conduction devices, contralateral routing of sound systems, and cochlear implants could be options for auditory rehabilitation. Although previous studies have shown a lack of beneficial effect of bone conduction devices or contralateral routing of sound systems regarding sound localization, speech perception is improved with these devices when speech is presented to the poorer ear [25, 26]. Single-sided deafness is now being considered as an indication for cochlear implantation and many studies have reported the benefits of cochlear implantation regarding sound localization, speech perception in noisy environments, and tinnitus [27, 28].

In conclusion, MHL is common in South Korea. It is associated with significant hearing problems, including subjective hearing discomfort, tinnitus, and poor quality of life. Nevertheless, hearing rehabilitation is extremely limited for patients with MHL. Therefore, minimally hearing-impaired patients, especially those with hearing handicaps, might be considered as candidates for auditory rehabilitation, including counselling regarding communication strategies and the option to evaluate the potential benefits of sound amplification.
